# Outcome a decade after laparoscopic and open Nissen fundoplication in children: results from a randomized controlled trial

**DOI:** 10.1007/s00464-022-09458-6

**Published:** 2022-08-01

**Authors:** Thomas J. Fyhn, Morten Kvello, Bjørn Edwin, Ole Schistad, Are H. Pripp, Ragnhild Emblem, Charlotte K. Knatten, Kristin Bjørnland

**Affiliations:** 1 Institute of Clinical Medicine, University of Oslo, Oslo, Norway; 2 Department of Gastrointestinal and Pediatric Surgery, Oslo University Hospital, 4950, Nydalen, Oslo, 0424 Norway; 3 The Intervention Centre, Oslo University Hospital, Oslo, Norway; 4 Department of Hepatopancreatobiliary Surgery, Oslo University Hospital, Oslo, Norway; 5 Oslo Centre of Biostatistics and Epidemiology, Research Support Services, Oslo University Hospital, Oslo, Norway; 6 Department of Pediatrics, Oslo University Hospital, Oslo, Norway

**Keywords:** Child, Fundoplication, Gastroesophageal reflux, Laparoscopy, Randomized

## Abstract

**Background:**

Randomized controlled trials (RCT) comparing long-term outcome after laparoscopic (LF) and open fundoplication (OF) in children are lacking. Here we report recurrence rates and time to recurrence, frequency of re-interventions, use of antisecretory drugs, gastrointestinal symptoms, and patient/parental satisfaction a decade after children were randomized to LF or OF.

**Methods:**

Cross-sectional long-term follow-up study of a two-center RCT that included patients during 2003–2009. Patients/parents were interviewed and medical charts reviewed for any events that might be related to the fundoplication. If suspicion of recurrence, further diagnostics were performed. Informed consent and ethical approval were obtained. Clinicaltrials.gov: NCT01551134.

**Results:**

Eighty-eight children, 56 (64%) boys, were randomized (LF 44, OF 44) at median 4.4 [interquartile range (IQR) 2.0–8.9] years. 46 (52%) had neurological impairment. Three were lost to follow-up before first scheduled control. Recurrence was significantly more frequent after LF (24/43, 56%) than after OF (13/42, 31%, *p* = 0.004). Median time to recurrence was 1.0 [IQR 0.3–2.2] and 5.1 [IQR 1.5–9.3] years after LF and OF, respectively. Eight (19%) underwent redo fundoplication after LF and three (7%) after OF (*p* = 0.094). Seventy patients/parents were interviewed median 11.9 [IQR 9.9–12.8] years postoperatively. Among these, use of anti-secretory drugs was significantly decreased from preoperatively after both LF (94% vs. 35%, *p* < 0.001) and OF (97% vs. 19%, *p* < 0.001). Regurgitation/vomiting were observed in 6% after LF and 3% after OF (*p* = 0.609), and heartburn in 14% after LF and 17% after OF (*p* = 1.000). Overall opinion of the surgical scars was good in both groups (LF: 95%, OF: 86%, *p* = 0.610). Patient/parental satisfaction with outcome was high, independent of surgical approach (LF: 81%, OF: 88%, *p* = 0.500).

**Conclusions:**

The recurrence rate was higher and recurrence occurred earlier after LF than after OF. Patient/parental satisfaction with outcome after both LF and OF was equally high.

**Graphical abstract:**

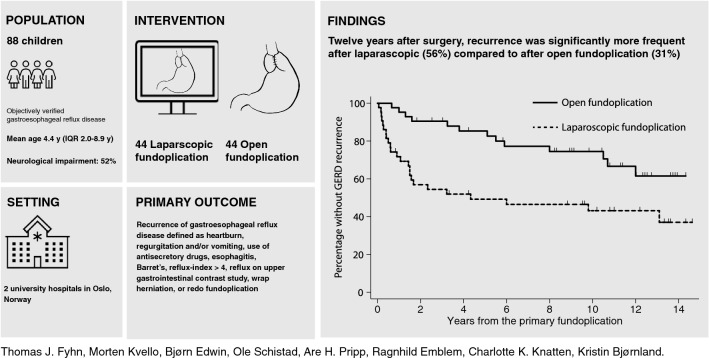

**Supplementary Information:**

The online version contains supplementary material available at 10.1007/s00464-022-09458-6.

Multiple randomized controlled trials (RCT) have shown that adults undergoing laparoscopic fundoplication (LF) have shorter convalescence, fewer complications, and better cosmetic results than those operated with open fundoplication (OF) [1]. Furthermore, these RCTs found no differences in recurrence rates of gastroesophageal disease (GERD) or patient satisfaction, even 10 to 15 years postoperatively [2, 3]. Consequently, LF has replaced OF as the surgical treatment of choice for adult GERD [4]. Pediatric surgeons have assumed that LF has the same benefits in children as in adults, and several non-randomized studies have shown better outcome after LF [5]. In children, only three RCTs including a total of 171 patients, have compared outcomes after LF and OF. A meta-analysis combining the results from these three RCTs reported higher recurrence rates and longer operation time after LF, but similar length of hospital stay and complication rates [6]. There are fundamental differences between children and adults undergoing anti-reflux surgery that may affect outcomes in unexpected ways. Around half of pediatric patients undergoing fundoplication have neurological impairment (NI) with diagnoses such as cerebral palsy and various syndromes [7]. NI is associated with gastrointestinal dysmotility that may influence sustainability of the fundoplication, and NI patients often have deteriorating health and increased mortality [8, 9]. Retching, common in NI patients, has strongly been linked to wrap breakdown [10]. Moreover, growth may influence the long-term durability of the fundoplication by causing strain on the wrap and/or adhesions, and the effects of growth may differ between open and laparoscopic surgery [11, 12]. Taken together, pediatric patients have many risk factors for recurrence of GERD, and recurrence rates up to 43% 10–15 years after fundoplication have been reported [13].

Since no RCT has compared outcome after LF and OF in pediatric patients a decade after fundoplication, we conducted this follow-up study in children randomized to LF and OF where we previously reported higher recurrence rates after LF median 4 years postoperatively [14]. The main aim was to explore whether the observed difference in recurrence rates between LF and OF persisted several years later. Furthermore, we wanted to compare time to recurrence, frequency of re-interventions, current gastrointestinal symptoms, patients’ assessment of the scars, and patient/parental satisfaction.

## Materials and methods

### Study design, participants, pre-, per- and postoperative protocol

This is a cross-sectional follow-up study of a two-center, prospective, randomized, non-blinded, parallel-group study comparing outcome after LF and OF in patients under 15 years at referral [14, 15]. Inclusion criteria were GERD confirmed by 24-h pH monitoring and/or upper gastrointestinal (UGI) contrast study and insufficient effect of conservative treatment. Exclusion criteria were comorbidity assessed incompatible with laparoscopy, multiple previous laparotomies and parents not speaking Norwegian. The trial was conducted at tertiary referral centers Oslo University Hospital Rikshospitalet and Ullevål. Randomization occurred between January 2003 and December 2009 and was performed by two of the authors (KB, RE) the day before surgery by using an opaque envelope containing five notes marked “LF” and five marked “OF”, thus blocks of 10 with 1:1 allocation ratio. To minimize risk of selection bias during preoperative information towards the end of each block, the next block of 10 was included when the current block had three remaining notes. No parents withdrew their child from the study after randomization.

Preoperative work-up and peri- and postoperative care were standardized as described in detail previously [14, 15]. All fundoplications were Nissen fundoplications and performed identically except for laparotomy or laparoscopy. This included division of some short gastric vessels, hiatal repair, and construction of a 360° loose wrap that was anchored to the esophageal wall and the diaphragm. All laparoscopies were done by consultants with experience from at least thirty LFs, whereas the OFs were either performed by consultants or trainees under consultant supervision. Adherence to the study protocol was monitored by the senior author (KB) who either performed or assisted at a majority of the procedures.

### Definitions

The primary outcome was rate of recurrent GERD after the two procedures. Recurrence was defined as any of the following; heartburn, regurgitation and/or vomiting > 1/week, use of anti-secretory drugs because of GER symptoms > 1/week, jejunal or parenteral feeding because of GERD, macroscopic esophagitis or new-onset Barrett’s esophagus, reflux-index > 4, GER demonstrated on UGI contrast study, wrap herniation found on UGI contrast study or by endoscopy, or redo fundoplication [16–18]. Time to recurrence was the number of years between the primary fundoplication and appearance of any criteria for recurrent GERD. NI means a static or progressive, central or peripheral neurological condition associated with intellectual disability and/or functional impairment [19].

### Evaluation of outcome

In the previous study, a clinical examination, an UGI contrast study and a 24-h pH monitoring were scheduled 6 months postoperatively, and then semi-structured telephone interviews were performed 1, 2, and 4 years postoperatively [14]. For this follow-up study on long-term outcome, semi-structured telephone interviews were conducted again by two of the authors (TJF, MK) between 2017 and 2019. Depending on the patients´ age and the ability to verbalize symptoms, the interviewers either talked with patients only, with parents only or with both patients and parents. If the patient lived in an institution, health care providers at the institution were also interviewed.

The frequency of the following symptoms were recorded: Typical GER symptoms (heartburn, vomiting, regurgitation), troublesome side effects of the fundoplication (dysphagia, bothersome flatulence, retching, discomfort during meals, early satiety), and usage of antisecretory drugs (proton pump inhibitors (PPI), H_2_-receptor antagonists). Furthermore, the ability to vomit was explored. We also asked if not being able to vomit caused the patient any discomfort or problems. Patients and/or parents were asked whether they were satisfied with the fundoplication, to compare present well-being with preoperative well-being, if they would opt for surgery again, and if they would recommend fundoplication to others in a similar situation. If both the patient and parents were interviewed, a consensus was reached. Lastly, assessment of the scar was recorded (Table 1).

**Table 1:** Questions for assessment of surgical scars after laparoscopic (LF) and open fundoplication (OF)

Question	LF (*n* = 19) Yes	OF (*n* = 22) Yes	*p* value
Has the scar been painful the past few weeks?	0% (0/19)	0% (0/22)	NA
Has the scar been itching the past few weeks?	5% (1/19)	9% (2/22)	1.000
Have you ever considered correctional surgery?	5% (1/19)	0% (0/22)	0.463
Have you ever seen a bulge or protrusion in or near the scar?	0% (0/19)	0% (0/22)	NA
Is the overall opinion of the scar compared to normal skin good?	95% (18/19)	86% (19/22)	0.610

Answers were registered as yes or no. Only patients able to understand the questions and communicate were asked

*NA* not applicable

A retrospective chart review was performed to record any new investigations or surgical reinterventions related to the primary fundoplication. If patients or parents during the telephone interview reported that new investigations or surgical reinterventions had been done at other hospitals, findings from these were obtained. Those who reported symptoms indicating recurrence or troublesome side effects, were invited to undergo appropriate investigations.

### Ethics

Participation was voluntary, and informed written consent was obtained. The study was approved by the Regional Committee for Medical Research Ethics (2018/919; S-03082) and is registered at https://clinicaltrials.gov (reference number NCT01551134).

### Statistical Analysis

**Fig. 1:**
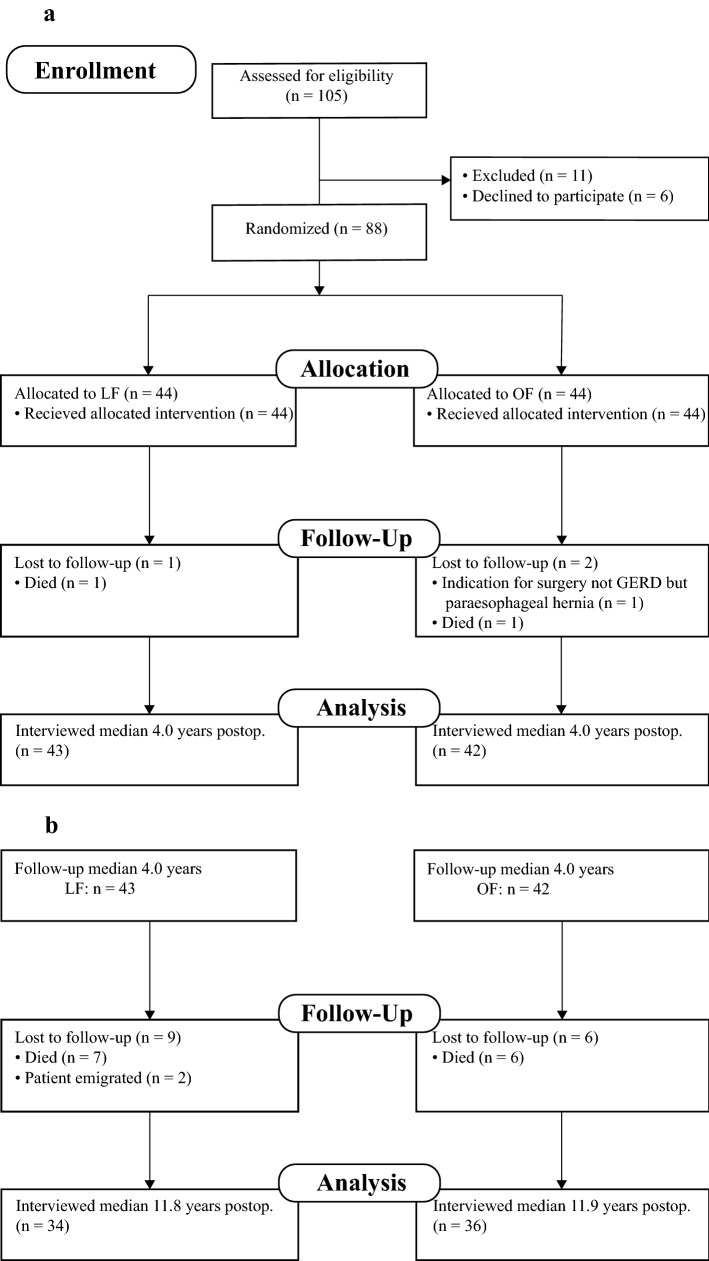
CONSORT flow chart for long time assessment of GERD recurrence in children randomized to open (OF) and laparoscopic (LF) fundoplication. **a** CONSORT flow chart for median 4.0 years follow-up.** b** CONSORT flow chart for median 11.9 years follow-up

Continuous data are presented as mean (standard deviation) if normally distributed or as median [interquartile range] if not normally distributed. Categorical data were analyzed using chi-square test or Fisher’s exact test, as appropriate, and expressed by risk ratio (RR) and 95% confidence interval (CI). The proportion of patients in the two groups using antisecretory drugs before and after the fundoplication was compared using the McNemar test. Kaplan–Meier plot and log-rank test were used to compare the occurrence of GERD recurrence, surgical reintervention and redo fundoplication between the two study groups. If a patient had more than one surgical reintervention, the first reintervention was selected as time of event. 85 patients were included in the Kaplan–Meier models and log rank analyses (Fig. 1a). Patients without recurrence/surgical reintervention/redo fundoplication who died or emigrated during the study period were censored at their last follow-up (right censoring, Fig. 1b). Univariate Cox regression analysis was performed for LF/OF to produce a hazard ratio and 95% CI for GERD recurrence/surgical reintervention/redo fundoplication. Data were analyzed according to intention-to-treat, thus all patients were analyzed in their original assigned group at primary fundoplication. Accordingly, patients originally assigned to and undergoing a primary LF fundoplication, were analyzed as part of the LF group, even if they later underwent an open re-fundoplication. We have previously described calculation of sample size and power [14]. Kaplan–Meier analyses was performed with STATA, version 15 (StataCorp, College Station, TX). All other statistical analyses were performed with IBM SPSS Statistics for Windows, version 27.0 (IBM, Armonk, NY). A *p* value of < 0.05 was considered statistically significant.

## Results

### Patients and completeness of follow-up

Of 105 eligible patients, 88 with a median age of 4.4 [2.0–8.9] years were randomized to LF or OF (Fig. 1A) [14, 15]. 46/88 (52%) were NI. The trial was terminated earlier than planned due to slow accrual of patients, as described previously [14]. Fifteen patients (17%) died during the follow-up period (LF: 8/44, 18%, OF: 7/44, 16%), median 7.2 [1.8–9.5] years (LF: 7.2 [0.8–9.1] years, OF: 5.6 [2.3–10.7] years) after the primary fundoplication. Mortality only occurred in NI patients. Two patients had emigrated. Evaluation for recurrence was possible in 85/88 patients (two patients died before first scheduled follow-up, and one patient was not operated for GERD) (Fig. 1A). Seventy patients and/or their parents agreed to participate (LF: 34, OF: 36) for this long-term follow-up study, and follow-up time for these was median 11.9 [9.9–12.8] years (Fig. 1B, Table 2).

**Table 2:** Demographics for 70 patients interviewed median 11.9 (interquartile range 9.8–12.8) years after laparoscopic (LF) and open fundoplication (OF)

	LF (*n* = 34)	OF (*n* = 36)
Age at interview, years, mean (SD)	17.5 (4.6)	16.7 (4.3)
Follow-up time, years, median [IQR]	11.8 [9.9–13.0]	11.9 [9.9–12.8]
Female, *n* (%)	16 (47)	11 (31)
Neurological impairment, *n* (%)	16 (47)	16 (44)
Gastrostomy at follow-up, *n* (%)	14 (41)	13 (36)
No comorbidity, *n* (%)	15 (44)	16 (44)

*SD* standard deviation, *IQR* interquartile range

### Recurrence of GERD

**Fig. 2:**
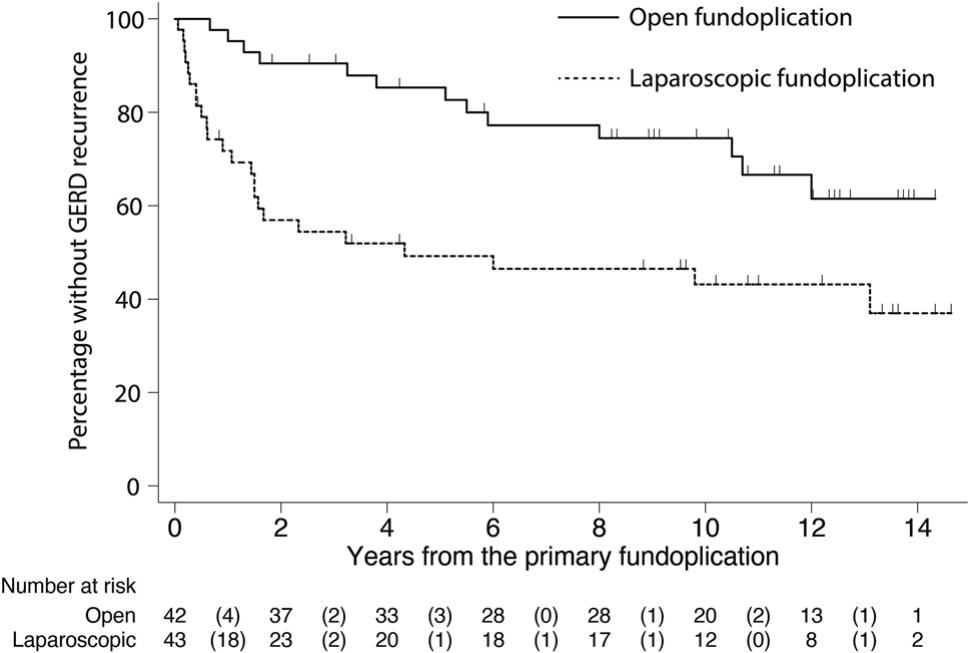
Kaplan–Meyer plot of gastroesophageal reflux disease (GERD) recurrence in patients randomized to laparoscopic or open fundoplication. The groups were compared using log-rank test. Tick marks indicate censored data

Recurrence was significantly more frequent (24/43, 56%) after LF than after OF (13/42, 31%, *p* = 0.004, Fig. 2). Furthermore, recurrence occurred earlier after LF (Fig. 2), median 1.0 [0.3–2.2] year after LF and 5.1 [1.5–9.3] years after OF. The hazard rate for recurrence after LF was 2.58 (95% CI 1.31–5.08, *p* = 0.006).

Of the patients categorized as having recurrence, objective findings of recurrence were demonstrated in 34/37 (92%) patients. A reflux-index > 4 and/or GER on upper GI contrast study was demonstrated more often after LF (15/43, 35%) than after OF [5/42, 12%, RR: 2.9 (95% CI 1.2–7.3), *p* = 0.012]. A herniated wrap was found on UGI contrast studies/esophagogastroscopy/computerized tomography in 23/85 (27%) of the patients [LF: 14/43, 33%, OF: 9/42, 21%, RR: 1.4 (95% CI 0.7–2.9), *p* = 0.306]. Three patients with recurrence had no objective verification of GERD (LF 1; OF 2). One of these refused testing, one had normal endoscopy while using PPI, and one had a normal pH study while using PPI. All three had typical GERD symptoms alleviated with PPI.

### Treatment of recurrent GERD and surgical reinterventions

Monotherapy with antisecretory drugs was the most common treatment modality for recurrent GERD and independent of surgical approach (Table 3). In addition, patients with recurrent GERD getting jejunal or parenteral feeding also used antisecretory drugs.

**Table 3:** Treatment of recurrent gastroesophageal reflux disease in pediatric patients randomized to laparoscopic (LF) or open fundoplication (OF)

	LF (*n* = 24)	OF (*n* = 13)
Only antisecretory drugs^a^	38% (9/24)	46% (6/13)
Re-fundoplication	33% (8/24)	23% (3/13)
Jejunal feeding and antisecretory drugs^a^	4% (1/24)	15% (2/13)
Parenteral feeding and antisecretory drugs^a^	8% (2/24)	0% (0/13)
No treatment	17% (4/24)	15% (2/13)

^a^Proton pump inhibitor or H_2_-receptor antagonists

Recurrent GERD initiated 21 surgical reinterventions in 16 patients (LF: 11/43, 26%, OF: 5/42, 12%, *p* = 0.069). Five patients had more than one reintervention. The hazard rate for surgical reintervention after LF was 2.57 (95% CI 0.89–7.41). Redo fundoplication was performed in 19% (8/43) and 7% (3/42) after LF and OF, respectively (*p* = 0.094). The hazard rate for redo fundoplication after LF was 2.95 (95% CI 0.78–11.12). The redo fundoplications were performed after median 1.8 [0.6–5.3] years. Five patients (all NI) got a transgastric jejunal tube (TGJ) to treat recurrent GERD. All first insertions of a TGJ were done under general anesthesia. Because of recurrent dislocations or unsatisfactory effect of the TGJ, four out of five patients reverted to other alternatives. One got a surgical jejunostomy, two a central venous catheter for parenteral nutrition, and one a redo fundoplication. The fifth patient is currently awaiting a surgical jejunostomy. Lastly, one patient underwent emergency surgery 10 years after LF due to incarceration of a herniated wrap. This patient underwent a gastrectomy due to gastric ischemia and got a Roux-en-Y esophagojejunostomy and a jejunostomy.

Six patients (two NI) with recurrence (four had wrap herniation, two had pathological pH index) did not get any anti-reflux treatment because symptoms were either very mild or absent. Symptoms in these patients presented were mild heartburn (two non-NI patients; LF: 1, OF: 1), regurgitation (one non-NI patient; LF), retching (one NI patient; OF). One none-NI and one NI patent did not have any symptoms in spite of herniation of the wrap (one patient) and a pathologic reflux-index (one patient). Both had undergone LF.

Overall opinion of the scars was good in most patients, and there was no difference in frequency of patients having considered correctional surgery for their scars (Table 1). Furthermore, no patients had experienced incisional or port site hernias or been operated for adhesion ileus.

### Symptoms at follow-up and satisfaction with the fundoplication

**Fig. 3:**
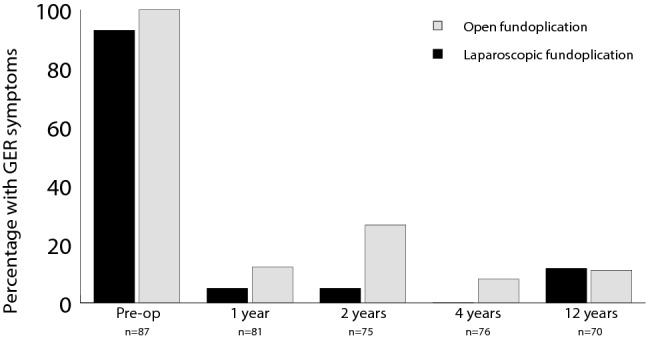
Percentage of patients with GER symptoms (heartburn, vomit, and/or regurgitation) before laparoscopic and open fundoplication, and at 1, 2, 4, and 12 years postoperatively. The number of patients/parents able to answer is indicated on the x-axis. Answers from patients with wrap herniation, who used antisecretory drugs and/or had surgical reintervention are also included

At follow-up, 62/70 patients (86%) had no GER symptoms, and there was no difference between the groups (Table 4, Fig. 3). There was a significant decrease in use of antisecretory drugs compared to preoperatively in both groups (LF: 94% vs. 33%, *p* < 0.001, OF: 97% vs. 19%, *p* < 0.001). At follow-up, there was no difference in the use of antisecretory drugs between the LF and OF group (*p* = 0.155).

**Table 4:** Patient/parental reported gastrointestinal symptoms occurring once a week or more often in patients median 11.9 (interquartile range 9.8–12.8) years after randomization to laparoscopic (LF) and open fundoplication (OF)

	LF (*n* = 34)	OF (*n* = 36)	Risk ratio (95% CI)	*p* value
Gastroesophageal reflux symptoms				
Heartburn	14% (3/21)	17% (4/24)	0.9 (0.2–3.4)	*p* = 1.000
Regurgitation/vomiting	6% (2/34)	3% (1/36)	2.1 (0.2–22.3)	*p* = 0.609
Other symptoms				
Retching	15% (5/34)	14% (5/36)	1.1 (0.3–3.3)	*p* = 1.000
Discomfort after meals	8% (2/26)	13% (4/31)	0.6 (0.1–3.0)	*p* = 0.678
Early satiety	19% (4/21)	19% (5/26)	1.0 (0.3–3.2)	*p* = 1.000
Bothersome flatus	40% (12/30)	31% (11/35)	1.3 (0.7–2.5)	*p* = 0.471
Dysphagia for solids	4% (1/23)	12% (3/25)	0.4 (0.0–3.2)	*p* = 0.610
Unable to vomit	53% (18/34)	75% (27/36)	0.7 (0.5–1.0)	*p* = 0.054
Perceived as a problem not being able to vomit	32% (11/34)	39% (14/36)	0.8 (0.4–1.6)	*p* = 0.568

Not all patients and/or parents were able to answer all questions

General well-being was better compared to preoperatively in almost all patients; 62/65 [LF: 94%, OF: 97%, *p* = 0.602, RR: 1.0 (95% CI 0.9–1.1)]. 62/65 (95%) would choose fundoplication again [LF: 90%, OF: 100%, *p* = 0.103, RR: 0.9 (95% CI 0.8–1.0)], and 64/65 (98%) would recommend fundoplication to others in a similar situation [LF: 97%, OF: 100%, *p* = 0.477, RR 1.0 (95% CI 0.9–1)]. All patients and parents who would not choose the same treatment again or recommend fundoplication to others, had symptomatic GERD recurrence.

Five adult patients were unable to answer questions on satisfaction with the fundoplication because they could not remember preoperative symptoms. Of the remaining 65 parents and patients, 55 were fully satisfied with the result of the operation [LF: 81%, OF: 88%, *p* = 0.500, RR 0.9 (95% CI 0.7–1.1)], six were partially satisfied (LF: 10%, OF: 9%), and one was unsure (LF). Three out of six partially satisfied responders listed symptomatic recurrence as reason for not being fully satisfied.

## Discussion

This study, the first RCT in children to compare outcome after LF and OF after a decade’s follow-up, finds that recurrence rate was higher and that recurrence occurred earlier after LF. Not only are RCTs comparing LF and OF very limited in the pediatric literature, but there are also very few retro- and prospective studies comparing long-term results after the two techniques. A retrospective study of 101 patients demonstrated equally good results after open and laparoscopic Thal fundoplication median 6.5 years postoperatively [20]. Other, non-comparative studies with a similar length of follow-up have found recurrence rates between 5 and 43% after LF and between 0 and 8% after OF [12, 13, 21–24]. It is difficult to explain the higher recurrence rate after LF, particularly since this is not the case in adults [2, 3]. As previously discussed, NI related gastrointestinal dysmotility, deteriorating health and growth may all contribute. Furthermore, it is possible that operations should not be performed identically minimally invasive and via open surgery. A RCT published after our study had started, compared minimal and extensive hiatal dissection during LF in children and showed that minimal hiatal dissection reduced the rate of wrap herniation [25]. All patients in our study underwent extensive hiatal dissection with division of the phrenoesophageal membrane and some short gastric as this was our standard method for OF. Supporting the hypothesis that minor modifications of an open operation when done minimally invasive may improve results, are recent recommendations for thoracoscopic repair of congenital diaphragmatic hernia [26, 27]. Since several reports showed higher recurrence rates after thoracoscopic repair, it is now advised to cauterize the edges of the defect in the diaphragm to promote scarring and thereby prevent recurrence [28–30]. Taken together, our and other studies on outcome of minimally invasive surgery in children, emphasize the importance of carefully studying results when new surgical techniques are introduced, even if similar operations have good results in adults.

Recurrence of GERD occurred earlier after LF than after OF. This finding is in line with results from a retrospective study including 450 pediatric patients undergoing LF or OF [31]. Non-comparative studies have reported that recurrence occurs on average 13–17 months after LF [32–34] and 10–21 months after OF [35–37]. We found that most recurrences after LF occurred during the first two years, whilst there was a more linear increase in recurrent GERD after OF. We have no apparent explanation for why recurrence occurred earlier after LF, but assume that the same mechanisms accounting for recurrence also influence the time of recurrence.

More than twice as many patients underwent a surgical reintervention after LF than after OF. This difference did not reach statistical significance, most likely because of too few patients in the two groups. The overall redo rate is in line with results from a RCT in adults with more than 10 years follow-up and with that of studies from pediatric patients with shorter observation times [31, 38, 39]. Nearly a fifth of those with recurrent GERD, was treated with post-pyloric feeding. Post-pyloric feeding through a gastrojejunal tube or a jejunostomy is an accepted treatment modality for children with severe feeding disorders as an alternative to both primary and redo fundoplication [40, 41].

Lower rates of adhesion ileus and incisional hernia have been suggested as important advantages of LF in children [42]. None of the patients in this study experienced adhesion ileus. Other studies in children report between one and five percent incidence of adhesion ileus after fundoplication, and more commonly after OF [35, 42–44]. Thus, our study supports that adhesion ileus is an uncommon complication after fundoplication in children. No incisional hernias were observed in patients in the present study, and this is in line with other pediatric series [35, 44, 45].

Overall satisfaction with the fundoplication was equally good in the two groups, and importantly, satisfaction was also high in those with recurrence. This corresponds with our previous findings median 4 years postoperatively, and with results from studies on children undergoing fundoplication with shorter observation times [14, 46–50]. It is interesting that patients/parents are satisfied despite recurrence. Based on comments from the telephone interviews, recurrent GER symptoms were often less severe than preoperative GER symptoms and could often be satisfactorily relieved with PPIs. Furthermore, some said they would choose surgery again despite recurrence because the patient had benefitted from the years without any GER symptoms.

LF leaves minimal scars, probably making LF more appealing to patients and caregivers. The literature shows that many patients and parents worry about scars [51]. A study reporting outcome after LF in children reported, as expected, a good cosmetic result 10 years postoperatively [21]. Surprisingly, we found that very few patients in the OF group were troubled by their scar. It is possible that we would have found more concerns about scars if more non-NI patients had been included. On the other hand, it may be that the benefit of surgery in terms of relief of GERD symptoms outweighed any concerns about scars.

More than half of the patients were unable to vomit, and this was more prominent after OF. Other studies have found that a similar fraction of patients is unable to vomit postoperatively [50, 52]. Some experienced not being able to vomit as a problem, for instance if sick with gastroenteritis or after binge drinking alcohol. Similar findings have been reported previously [21, 53]. That the inability to vomit persists for at least a decade and that this may be uncomfortable in certain situations, should be included in preoperative information. In line with this, it is also important to notify patients about continued bothersome flatulence as about one third of the patients reported this as an inconvenient side effect of the fundoplication.

Dysphagia was uncommon in this study, and none was reoperated or underwent endoscopic dilatation because of postoperative dysphagia. Others have found that 0–24% of children need endoscopic dilatation after fundoplication [44, 54–56]. It is possible that our patients did not need any intervention due to dysphagia because we routinely checked the tightness of wrap and in many also had a bougie in the esophagus while the wrap was constructed. However, there is a possibility that dysphagia was unrecognized in patients with a gastrostomy and in those who could not verbalize complaints. In adults, dysphagia after fundoplication is common with 33% reporting mild dysphagia 10 years after LF and OF [38]. Esposito et al. reported mild dysphagia in 28% of non-NI children 10 years after LF [21]. Different ways of recording dysphagia may explain the different results. We only registered dysphagia for solids and did not grade dysphagia.

We report one life threatening complication in this series; gastric ischemia because of incarceration of a herniated wrap. This is a very uncommon complication [57, 58]. If a herniated wrap is left untreated, either because it does not cause any symptoms or the patient is very frail, it is of crucial importance to inform patients and parents that incarceration, although extremely rare, is a life-threatening complication. Furthermore, adult health care providers may not be aware of the possibility of late recurrence and may interpret symptoms of recurrence and wrap incarceration wrongly. Particularly in NI patients, it is challenging to diagnose GERD [59]. Therefore, we would strongly advice physicians to inform patients and parents about the possibility of long-term recurrence.

Strengths of this study include the long follow-up period, the RCT design, and that all patients had a meticulous follow-up by study personnel not involved in treating the patients. The patient population is heterogeneous including patients with various comorbidities, like most pediatric series reporting outcome after fundoplication. Therefore, we believe our results are generalizable. It would have strengthened the conclusions if all patients had undergone new investigations to detect recurrence. However, since all patients were followed prospectively at several time points, it is unlikely that symptomatic recurrence was undiagnosed. Lastly, it would have strengthened the study if validated questionnaires for evaluating GERD and health related quality of life had been included.

In conclusion, this RCT shows a higher rate of recurrent GERD and that recurrence occurs earlier after LF than after OF. Independent of surgical approach, patient, and parental satisfaction remains high more than 10 years after the fundoplication. The results from our study are important for the process of shared decision making when deciding how to perform antireflux surgery in children and emphasize the need to study results after introduction of new surgical techniques in children.

## Supplementary Information

Below is the link to the electronic supplementary material.

Supplementary file1 (DOC 218 kb)
